# Determinants of poor glycemic control among adult patients with type 2 diabetes mellitus in Jimma University Medical Center, Jimma zone, south west Ethiopia: a case control study

**DOI:** 10.1186/s12902-019-0421-0

**Published:** 2019-08-29

**Authors:** Yitagesu Mamo, Fekede Bekele, Tadesse Nigussie, Ameha Zewudie

**Affiliations:** 1 Department of Pharmacy, College of medicine and health science, Mizan Tepi University, Mizan Teferi, Ethiopia; 2 Department of Pharmacy, Jimma University Institute of Health Science, Jimma, Ethiopia; 3 Department of Public Health, College of medicine and health science, Mizan Tepi University, Mizan Teferi, Ethiopia; 4 Department of Pharmacy, College of medicine and health science, Mizan Tepi University, Mizan Teferi, Ethiopia

**Keywords:** Determinants, Diabetes mellitus, Glycemic control, Jimma university medical center

## Abstract

**Objective and background:**

In 2015 approximately 5.0 million people were estimated to have died from diabetes. Poor glycemic control is the most determinant of diabetes-related complication and death. The percentage of patients whose blood glucose level are not well controlled remains high yet. The aim of this study is to identify the determinants of poor glycemic control at the diabetes clinic of the Jimma University Medical Center from April 01 to June 30/2017.

**Methods:**

Facility-based case-control study design was conducted on patients with type 2 diabetes mellitus on follow-up at the diabetes clinic of Jimma University medical center. The consecutive sampling technique was employed and data were collected from April to June 2017. The data were entered using Epidata manager version 4.0.2 and exported to SPSS Version 21 for analysis. Logistic regression analysis was performed and variables with the *p*-value of less than 0.05 were considered as statistically significant determinants of poor glycemic control.

**Result:**

The study was conducted on 410 patients, of which 228 males and 182 females. The determinants of poor glycemic control were comorbidities [Adjusted odd ratio(AOR) = 2.56, 95%CI = 1.10–5.96], lack of self-monitoring blood glucose [AOR = 3.44,95%CI = 1.33–8.94], total cholesterol level of 200 mg/dl or more [AOR = 3.62, 95%CI = 1.46–8.97], diabetes duration of greater than 7 years [AOR = 3.08, 95%CI = 1.33–7.16], physical activity of three or less than three days [AOR = 4.79, 95%CI = 1.70–13.53], waist to hip ratio of 0.9 or greater for male and 0.85 or greater for female [AOR = 3.52, 95%CI = 1.23–10.11], being on metformin plus insulin [AOR = 9.22, 95%CI = 2.90–29.35] and being on insulin [AOR = 4.48, 95%CI = 1.52–13.16].

**Conclusion:**

Lack of Self-monitoring blood glucose, presence of comorbidities, duration of diabetes mellitus, physical activity of three or less than three days, total cholesterol of 200 mg/dl or more, waist to hip ratio of 0.9 or greater for male and 0.85 or greater for female, and types of antidiabetic medication were the independent predictors of poor glycemic control. Effort should be made towards reducing these factors by the concerned body.

## Background

Diabetes is among the largest and rapidly growing global health emergencies of the twenty-first century. Worldwide about 415 million adults are living with diabetes and it is projected to be 642 million in 2040 [1].

A major concern in the management of diabetes is the occurrence of complications. Complications in diabetes are related to the damaging effects of hyperglycemia. The major long-term diabetes complications are macrovascular (peripheral arterial disease, stroke, and coronary artery disease); and microvascular (retinopathy, neuropathy, and nephropathy [2]. Clinical trials have demonstrated that tight blood glucose control correlates with a reduction in those complications in a patient with type 2 diabetes mellitus (T2DM) [3, 4]. Another study also revealed that intensive glucose control reduced risk for some cardiovascular disease like; nonfatal myocardial infarction in T2DM but did not reduce risk for cardiovascular or all-cause mortality [5].

Diabetes mellitus created a great health and economic burden because of the direct costs of treatment, man-hours lost due to the debilitating effect the disease on the individual and society at large in the world [6, 7]. In 2015, approximately 5.0 million people were estimated to have died from diabetes [8]. In many urban areas of Sub-Saharan Africa, diabetes is as higher as or higher than, those in most Western European countries [9, 10].A study shows that 5% of adult deaths in Addis Ababa, Ethiopia were attributed to diabetes [11].

Glycemic control is the common factor that determines death and complication from diabetes [12, 13]. The risk of complication in type 2 DM is directly related to prior glucose control level. A study revealed that in patients with type 2 diabetes, HbA(1c) levels were associated with lower risks of macrovascular events and death down to a cutpoint of 7.0% while microvascular events down to a cutpoint of 6.5% [14].

As many factors affect blood glucose level, reasons for poor glycemic control is multifactorial and complex.. Factors like; a delay in the beginning and intensification of insulin unnecessarily, poor adherence to treatment, diet and exercise affect glycemic control [15].

In addition to these, another study shows that in patients with type 2 diabetes short disease duration and treatment with few oral glucose-lowering drugs were predictors of blood glucose level. Moreover, it also indicated that in developing nations; patients, doctors and health service factors, all affect glycemic targets [16]. Yet, another study revealed age and length of time patient lived with DM, receiving monotherapy compared with the combination of insulin and oral antidiabetics were more likely associated with good glycemic control, and self-management behavior did not appear to influence glycemic control [17].

In Ethiopia, a number of studies were conducted on factors affecting glycemic control, in different hospital of the country. Two of them indicated that younger age, hypertension, and non-adherence to diabetes self-management behaviors were independent predictors of poor glycemic control [18, 19]. In similar ways, a cross-sectional study done in Jimma University Medical center (JUMC) indicated that patients who have no formal education and farmer, taking a combination of insulin and oral medication, and poor adherence to the medication associated with poor glycemic control [20].

However, changeable cardiovascular risk factors such as dyslipidemia, obesity, physical inactivity, smoking were not studied well in patients with diabetes in Ethiopia. A little has been done on dyslipidemia from cardiovascular risk factor as a factor contributing to glycemic control in Addis Ababa [19], and only hypertension from adjustable cardiovascular risk factor was investigated as a factor contributing to glycemic control in JUMC [20]. The studies previously conducted in Ethiopia were cross-sectional [18, 20, 21]. This study was a case-control; besides the modifiable cardiovascular risk factors associated with glycemic control was also investigated.

## Objectives of the study

- To identify the predictors of poor glycemic control among adult patients with type 2 diabetes mellitus on follow up at the diabetic clinic of JUMC, 2017.

## Methods

### Study setting and period

This study was conducted from April 01 to June 30, 2017, at the diabetic clinic of JUMC. Geographically, it is located in Jimma city, 352 km southwest of Addis Ababa, the capital city of Ethiopia**.** JUMC is the only teaching hospital in southwest Ethiopia, providing services for approximately 15,000 inpatients, 160,000 outpatient attendants, 11,000 emergency cases and 4500 deliveries annually with the catchment population of about 15 million people [http://www.ju.edu.et/jimma-universityspecialized hospital-jush].

### Study design and population

Facility based-case control study was conducted. The source population was all type 2 diabetes mellitus. The study population was all patients with type 2 diabetes mellitus who presented at a diabetic clinic during the data collection period and those who fulfilled the eligibility criteria. **Cases** were patients with type 2 diabetes mellitus who had poor glycemic control and **Controls** were patients with type 2 diabetes mellitus who had good glycemic control.

### Inclusion criteria

Patients with type 2 diabetes mellitus whose age were greater than 18 years and who had at least three months consecutive follow up were included in both cases and control.

### Exclusion criteria

In both cases and controls, patients with type 2 diabetes mellitus who were mentally unstable or critically ill and who were not able to respond were excluded.

### Sample size and sampling technique

#### Sample size

Epi info version 7 was used to calculate sample size by two population proportions with the assumption of 95% Confidence Interval (CI), 80% power, and 1:1 case to controls ratio. Odds Ratio (OR) and proportion of different predictor variable of glycemic control among controls were taken from a study done at Mekelle, Ambo, and Jimma [18, 20, 21]. Then the largest total sample size became 410. Since the ratio of case to control was 1:1, the sample size was calculated to be 205 for both cases and controls**.**

#### Sampling technique

All patients with type 2 diabetes mellitus attending the diabetic clinic during the working time of the clinic and eligible were enrolled. Consecutive sampling technique was applied to recruite the required sample sizes of both groups (cases or controls) were achieved. Study participants were interviewed up on their exit from diabetic clinic.

### Data collection procedures and instruments

Data were collected using a structured questionnaire which was developed based on different literatures [18, 20–24]. The questionnaire was translated from English to Afan Oromo and Amharic and back-translated to English. Checklist was used to collect data related to patients’ medications and laboratory parameters.

### Data collection process and management

Information related to socio-demographic characteristics, self-care activities and medication adherence were collected through interviews with the patients. Weight, height, Hip Circumference (HC) and Waist Circumference(WC) were measured during the day of the interview. Three consecutive months Fasting Blood Glucose (FBG), latest Fasting Lipid Profile (FLP) laboratory values within one year and other clinical characteristics were obtained from patients’ records.

### Study variables

Dependent variable was the **status of glycemic control.** Independent variables include **socio-demographic variables** (age, sex, marital status, religion, educational status, income and occupation), **diabetes self-care factors** (Self-Monitoring Blood Glucose (SMBG), adherence to antidiabetic medication, knowledge of target blood sugar, attendance diabetic education program, alcohol consumption, khat chewing, adherence to healthy eating plan and cigarette smoking), **clinical related factors** (duration of diabetes, comorbidities, Waist to Hip Ratio(WHR), Body Mass Index(BMI) and FLP) and **medication-related factors(**types of antidiabetic medication and polypharmacy).

### Operational definitions and measurements

**Glycemic control**: patients were categorized based on the American Diabetic Association (ADA) 2017 guideline recommendation [25] into two groups:

**Good glycemic control**: average fasting blood glucose of 80–130 mg/dL.

**Poor glycemic control:** average fasting blood glucose of > 130 mg/dL.

**Knowledge of target blood glucose:** was assessed by use of “yes/no” questions. Mentioning correct answer was coded as “1” and failure to mention as “0” Then the score was converted to a percentage score. The mean score was used to classify patients into adequate and inadequate knowledge level.

**Adherence to diet**: If the respondents follow a recommended diet for more than 3 days in last seven days.

**Adherence to exercise:** If the respondents follow the recommended level of exercise for more than 3 days in the last seven days.

**Fasting blood sugar**: blood glucose measured from venous blood after at least 8 h of overnight fasting.

**Adherence to medication**: if the patients took all his/her antidiabetic medication in the last seven days.

**Alcohol consumption**- if reported consumption of alcohol twelve-month prior to the survey.

**Polypharmacy:** taking more than four medications daily. Simultaneous polypharmacy: was used to estimate the number of drugs a patient is receiving at any given point in time [26].

**BMI of 25.0–29.9 kg/m2** -were classified as overweight and BMI of ≥30.0 kg/m2- were classified as obese [27]. **Abdominal obesity**- Waist to Hip Ratio (WHR) ≥ 0.90 for males and ≥ 0.85 for females [28].

### Measurements

After the participants stood with arms at the sides, feet positioned close together, and weight evenly distributed across the feet, the WC was measured to the nearest 1 cm three times at the approximate midpoint between the lower margin of the last palpable rib and the top of the iliac crest, at the end of a normal exhalation. The mean of the three measurements was calculated and taken at the end. Participants told to relax and take a few deep, natural breaths before the actual measurement was made in order to minimize the inward pull of the abdominal contents during the waist measurement. Participants have already fasted overnight to measure their FBG. This condition was also beneficial for measuring WC. HC of the patients was measured three times to the nearest centimeter at the largest circumference of the buttocks. Both hip and waist circumference were measured with a stretch-resistant tape that is wrapped snugly around the participants and the tape was kept level and parallel to the floor at the point of measurement. This protocol of measurement was per WHO STEPwise approach to surveillance [29]. Height was measured to the nearest centimeter with respondents standing on a hard surface against a wall, using a square and tape measure to the wall. All measurements were recorded to the nearest centimeter. Weight was measured to the nearest 100 g using a calibrated instrument. WHR was calculated as WC (cm) divided by HC (cm). BMI was calculated as the weight divided by height squared (kg/m2).

### Data processing and analysis

The data on the questionnaire was entered into Epidata manager version 4.0.2 and double entry verification was made and exported to SPSS version 21 statistical packages for analysis. The data were explored to check outliers, missing data and assumptions. During analysis frequencies of the different variables were determined as necessary; Cross-tabulations and bivariate analysis were performed to select variables for multivariate analysis. Hence variables with a *p*-value < 0.25 in the bivariate analysis were taken as candidates for multivariable analysis. Finally, multivariable logistic regression analysis was performed to control for the possible confounding effect of the selected variables and variables with a *p*-value of less than 0.05 were taken as statistically significant predictors for poor glycemic control and OR with its 95% CI was used to show the degree of association between the independent and the outcome variable.

### Data quality assurance

The structured questionnaire was adapted from different related studies. Then it was translated from English to Afan Oromo and Amharic and back-translated into English by the independent person to assure its consistency. Three days training was given for three pharmacists (Bpharm) and 2 nurses (Bsc. Nurse) prior to data collection. The pharmacists collected the data through patient interview and from the card, whereas nurses measured the waist circumference, hip circumference, height and weight of the respondents. A panel of experts (clinical pharmacists) assessed whether the data collection form would measure what it intended to measure and if it was comprehensive enough to collect all the information needed to address the purpose and goals of the study. Then pilot test was done on 21 patients(5%) and necessary changes were made based on expert opinion.

## Result

### General characteristics of the study participants

This study was conducted on 410 patients of type 2 diabetes mellitus, 205 cases, and 205 controls, of which 54 and 58% were males respectively. The mean ± SD age of the respondents were 52.63 ± 10.46 and 53.33 ± 11.73% of cases and controls respectively. More than one-third of both cases and controls were in the age group “45–54 years”. Seventy four percent of cases and 85% of control were married. Thirty four percent and 20.5% of cases and controls, respectively, had no formal education (Table 1).

**Table 1:** Socio-demographic characteristics of adult patients with type 2 diabetes mellitus on follow up at the diabetic clinic of Jimma University medical center, south-west Ethiopia, April 01–June 30, 2017

Variables	Cases N (%)	Control N (%)	Total N (%)
Age category			
25–34	8(3.90)	9 (4.40)	17 (4.10)
35–44	36 (17.60)	35 (17.10)	71 (17.30)
45–54	75 (36.60)	69 (33.70)	144 (35.10)
55–64	57 (27.80)	60 (29.30)	117 (28.50)
65 and above	29 (14.10)	32 (15.60)	61 (14.90)
Gender			
Male	110 (53.70)	118 (57.60)	228 (55.60)
Female	95 (46.30)	87 (42.40)	182 (44.40)
Religion			
Muslim	124 (60.50)	116 (56.60)	240 (58.50)
Orthodox	59 (28.80)	67 (32.70)	126 (30.70)
Protestant	20 (9.80)	17 (8.30)	37 (9.00)
others^a^	2 (0.97)	5 (2.40)	7 (1.70)
Ethnicity			
Oromo	119 (58.00)	120 (58.50)	239 (58.30)
Amara	40 (19.50)	34 (16.60)	74 (18.00)
Kaficho	8(3.90)	10 (4.90)	18 (4.40)
Dwuro	6 (2.90)	7 (3.40)	13 (3.20)
Yem	14 (6.80)	16 (7.80)	30 (7.30)
Gurage	12 (5.90)	4 (2.00)	16 (3.90)
Others^b^	6 (2.90)	14 (6.80)	20 (4.90)
Marital status			
Married	151 (73.70)	173 (84.40)	324 (79.00)
Single/divorced /widowed	54 (26.30)	32 (15.60)	86 (21.00)
Place of residence			
Rural	122 (59.50)	73 (35.60)	195 (47.60)
Urban	83 (40.50)	132 (64.40)	215 (52.40)
Educational status			
No education	69 (33.70)	42 (20.50)	111 (27.10)
Primary	71 (34.60)	66 (32.20)	137 (33.40)
Secondary	35 (17.10)	51 (24.90)	86 (21.00)
Tertiary	30 (14.60)	46 (22.40)	76 (18.50)
Occupational status			
Farmer	107 (52.20)	73 (35.60)	180 (43.90)
Employee	63 (30.70)	85 (41.50)	148 (36.10)
Merchant	9 (4.40)	16 (7.80)	25 (6.10)
Private	7 (3.40)	6 (2.90)	13 (3.20)
Housewife	8(3.90)	16 (7.80)	24 (5.90)
Others	11 (5.40)	9 (4.40)	20 (4.90)
Income			
< 1500	104 (50.70)	114 (55.60)	218 (53.20)
1500–600	55 (26.80)	63 (30.70)	118 (28.80)
≥ 6000	7 (3.40)	5 (2.40)	12 (2.90)
No constant income	39 (19.00)	23 (11.20)	62 (15.10)

^a^ Catholic and wake data, kambata, ^b^ walayita, Tigre and Silte, ^c^ Has not employed, retired and daily labor

### Diabetes self-care activities

Forty seven (22.90%) of cases and 65(31.70%) of controls had adequate healthy eating plan during the previous week before the study. Twenty two percent of the cases and 45% of the controls have involved in, at least 30 min of, physical activity for more than 3 days during the last seven days preceding the study (Table 2).

**Table 2:** Diabetes self-care activities of adult patients with type 2 diabetes mellitus on follow up in a diabetic clinic of Jimma University medical center, south-west Ethiopia, April 01–June 30, 2017

Variables	Cases, N (%)	Control, N (%)	Total N (%)
Know optimum blood sugar			
No	123 (60.00)	129 (62.90)	252 (61.50)
Yes	82 (40.00)	76 (37.10)	158 (38.50)
Diabetes education			
Yes	139 (67.80)	143 (69.80)	282 (68.80)
No	66 (32.20)	62 (30.20)	128 (31.20)
Physical activity			
> 3 days(adequate)	44 (21.50)	92 (44.90)	136 (33.20)
0–3 days (in adequate)	161 (78.50)	113 (55.10)	274 (66.80)
Self-monitoring blood glucose			
Yes	41 (20.00)	61 (29.80)	102 (24.90)
No	164 (80.00)	144 (70.20)	308 (75.10)
Smoke cigarette			
Yes, now	3 (1.50)	0 (0.00)	3 (0.70)
Yes, previousily	6 (2.90)	12 (5.90)	18 (4.40)
No never	196 (95.60)	193 (94.10)	389 (94.90)
Five or more serving of fruits per week			
> 3 days	23 (11.20)	20 (9.80)	43 (10.50)
0-3 days	182 (88.80	185 (90.20)	367 (89.50)
Compliance with general healthy eating plan			
0–3 days (inadequate)	158 (77.10)	149 (72.70)	307 (74.90)
> 3 days (adequate)	47 (22.90)	56 (27.30)	103 (25.10)
Chew khat			
Yes, now	85 (41.50)	72 (35.10)	157 (38.30)
Yes, previously	54 (26.30)	75 (36.60)	129 (31.50)
No	66 (32.20)	58 (28.30)	124 (30.20)
Alcohol consumption			
Yes	27 (13.20)	39 (19.00)	66 (16.10)
No	178 (86.80)	166 (81.00)	344 (83.90)

### Types of antidiabetic therapy and polypharmacy

Thirty six percent of cases were prescribed with Metformin plus Glibenclamide followed by insulin alone (31.70%). In controls, 31.20% were prescribed with insulin alone followed by metformin alone (29.80%) (Table 3). Twenty four percent (23.90%) of cases and 10.20% of control were taking more than or equal to five medications including medication for blood glucose control (Table 3).

**Table 3:** Biochemical parameters and clinical characteristics of adult patients with type 2 diabetes mellitus on follow up at the diabetic clinic of Jimma University medical center, south-west Ethiopia, April 01–June 30, 2017

Variables	Cases, N (%)	Controls, N (%)	Total, N (%)
Total cholesterol			
< 200 mg/dL	45 (58.40)	65 (81.30)	110)70.10)
≥ 200 mg/dL	32 (41.60)	15 (18.80)	47 (29.90)
Triglyceride			
< 150	43 (58.10)	49 (64.50)	92 (61.30)
≥ 150	31 (41.90)	27 (35.50)	58 (38.70)
HDL			
M: ≥40 mg/Dl, F:≥50 mg/dL	44 (60.30)	42 (55.30)	86 (57.70)
M:< 40 mg/dL, F:< 50 mg/dL	29 (39.70)	34 (44.70)	63 (42.30)
LDL			
< 100 mg/dL	53 (68.80)	60 (76.90)	113 (72.90)
≥ 100 mg/dL	24 (31.20)	18 (23.10)	42 (27.10)
BMI			
< 25	131 (63.90)	129 (62.90)	260 (63.40)
25–29.99	56 (27.30)	60 (29.30)	116 (28.30)
≥ 30	18 (8.80)	16 (7.80)	34 (8.30)
Waist to hip ratio			
M:< 0.9, F:< 0.85	20 (9.80)	64 (31.20)	84 (20.50)
M: ≥0.9, F: ≥0.85	185 (90.20)	141 (68.80)	326 (79.50)
Duration			
≤ 7 years	98 (47.80)	141 (68.80)	239 (58.30)
> 7 years	107 (52.20)	64 (31.20)	171 (41.70)
Types of antidiabetes medication			
Glibenclimide alone	4 (2.00)	3 (1.50)	7 (1.71)
Metformin+Glibenclimide	74 (36.10)	49 (23.90)	123 (30.00)
Insulin alone	65 (31.70)	64 (31.20)	129 (31.46)
Metformin + Insulin	33 (16.10)	28 (13.70)	61 (14.88)
Metformin alone	29 (14.10)	61 (29.80)	90 (21.95)
Polypharmacy			
Yes	49 (23.90)	21 (10.20)	70 (17.10)
No	156 (76.10)	184 (89.80)	340 (82.90)

### Selected clinical characteristics

The duration of diabetes was greater than 7 years in 52.20% of the cases and 31.20% of the controls. Ninty percent (90.20%) of the cases and 68.8% of the controls had WHR of ≥0.9 for males or ≥ 0.85 for females. From a total of 157(77 cases and 80 controls) participants for whom laboratory fasting lipid profile of total cholesterol were recorded. The mean total cholestrol was 193+/− 11 in cases and 171 +/− 14 in controls. Forty two percent(41.60%) of the cases as compared to 18.80% of the control had total cholesterol level of ≥200 mg/dl (Table 3).

### Predictors of poor glycemic control

In bivariate logistic regression, marital status, educational level, place of residence, self-monitoring blood glucose, physical activities, comorbidity, duration since diagnoses of diabetes, adherence to antidiabetic medication during the week preceding the study, polypharmacy, types of antidiabetic medication, WHR and total cholesterol level were found to be associated with poor glycemic control and entered into multivariate logistic analysis. In multivariate logistic analysis, self-monitoring of blood sugar, duration since diagnosis of diabetes, physical activity, types of diabetes medication, comorbidities, total cholesterol level and WHR were found to be significantly associated with poor blood sugar control as depicted in Table 4.

**Table 4:** Bivariate and multivariate logistic regression of determinants of poor glycemic control among adult patients with type 2 diabetes mellitus in Jimma University Medical Center, April 01–June 30, 2017

Variables	Cases	Control	Crude OR	Adjusted OR
Educational status				
No education	69 (33.70)	42 (20.50)	2.52 (1.38–4.58)	2.78 (0.54–14.41)
Primary	71 (34.60)	66 (32.20)	1.65 (0.93–2.91)	1.33 (0.36–4.94)
Secondary	35 (17.10)	51 (24.90)	1.05 (0.56–1.97)	0.72 (0.18–2.80)
Tertiary	30 (14.60)	46 (22.40)	1	1
Place of residence				
Rural	122 (59.50)	73 (35.60)	2.66 (1.78–3.96)	0.66 (0.24–1.85)
Urban	83 (40.50)	132 (64.40)	1	1
Comorbidity				
Yes	138 (67.30)	116 (56.60)	1.58 (1.06–2.36)	• 2.56 (1.10–5.96) *
No	67 (32.70)	89 (43.40)	1	1
Self-monitoring Blood Glucose				
No	164 (80.00)	144 (70.20)	1.69 (1.07–2.67)	3.44 (1.33–8.94)^*^
Yes	41 (20.00)	61 (29.80)	1	1
Total cholesterol				
≥ 200 mg/dL	32 (41.60)	15 (18.80)	3.08 (1.50–6.34)	3.62 (1.46–8.97) ^*^
< 200 mg/dL	45 (58.40)	65 (81.30)	1	1
Adherence to antidiabetic medication				
No	56 (27.30)	33 (16.10)	1.96 (1.21–3.17)	0.67 (0.26–1.69)
Yes	149 (72.70)	172 (83.90)	1	1
Duration of diabetes				
> 7 years	107 (52.20)	64 (31.20)	2.40 (1.61–3.60)	3.08 (1.33–7.16) *
≤ 7 years	98 (47.80)	141 (68.80)	1	1
Physical activity				
0-3 days (inadequate)	161 (78.50)	113 (55.10)	2.98 (1.93–4.60)	4.79 (1.70–13.53) *
> 3 days(adequate)	44 (21.50)	92 (44.90)	1	1
Polypharmacy				
Yes	49 (23.90)	21 (10.20)	2.75 (1.58–4.79)	1.41 (0.37–5.41)
No	156 (76.10)	184 (89.80)	1	1
Marital status				
Single/divorced/widowed	54 (26.30)	32 (15.60)	1.93 (1.19–3.15)	1.80 (0.68–4.72)
Married	151 (73.70)	173 (84.40)	1	1
Waist to hip ratio				
M: ≥0.9, F: ≥0.85	185 (90.20)	141 (68.80)	4.20 (2.43–7.26)	3.52 (1.23–10.11) *
M:< 0.9, F:< 0.85	20 (9.80)	64 (31.20)	1	1
Types of antidiabetes medication				
Glibenclimide alone	4 (2.00)	3 (1.50)	2.80 (0.59–13.36)	3.57 (0.18–68.66)
Metformin+Glibenclimide	74 (36.10)	49 (23.90)	3.18 (1.80–5.62)	9.22 (2.90–29.35) ^*^
Insulin alone	65 (31.70)	64 (31.20)	2.14 (1.22–3.74)	4.48 (1.52–13.16) ^*^
Metformin + Insulin	33 (16.10)	28 (13.70)	2.48 (1.27–4.84)	3.73 (0.87–16.05)
Metformin alone	29 (14.10)	61 (29.80)	1	1

M-Male, F-Female, OR-odd ratio, ^*^ Determinants of poor glycemic control

Participants who had comorbidity/ies were 2.56 times more likely to have poor blood glucose control compared to those who had no comorbidity/ies (AOR = 2.56, 95%CI = 1.10–5.96). Respondents who were not self-monitoring their blood glucose were 3.44 times more likely to have poor glycemic control as compared to those who were monitoring their blood glucose level(AOR = 3.44,95%CI = 1.33–8.94). Respondents who had total cholesterol of ≥200 mg/dl were 3.62 times more likely to have poorly controlled blood glucose compared to those who had total cholesterol of less than 200 mg/dl (AOR = 3.62, 95%CI = 1.46–8.97). Respondents who were diagnosed with diabetes more than seven years ago were 3.08 times more likely to have poorly controlled blood glucose than respondents diagnosed with diabetes less than or equal to seven years ago (AOR = 3.08, 95%CI = 1.33–7.16).

Respondents who do 30 min physical activity for 1 to 6 days were 4.79 times more likely to have poorly controlled blood glucose compared to those who do 30 min physical activity daily (AOR = 4.79, 95% CI = 1.70–13.53).

Participants who had a WHR of “≥0.9” for male or ≥ 0.85″ for female were 3.52 more likely to have poorly controlled blood glucose level as compared to participants who have a WHR of “< 0.9” for male or “< 0.85” for female (AOR = 3.52, 95%CI = 1.23–10.11).

Those diabetes patients who were on the combination of metformin and glibenclamide were 9.22 times more likely to have poorly controlled blood glucose compared to those patients who were on metformin alone (AOR = 9.22, 95%CI = 2.90–29.35). Similarly, patients who were on insulin alone were 4.48 times more likely to be poorly controlled than patients who were on metformin alone (AOR = 4.48, 95%CI = 1.52–13.16).

## Discussion

Glycemic control is the major therapeutic goal for prevention of organ damage and related complication of diabetes [30]. The American Diabetes Association recommends an HbA1c level of below 7% as a target for optimal blood glucose control and further recommended adequate glycemic control with pre-prandial capillary plasma glucose 80–130 mg/dl [25].

This study showed that high WHR, total cholesterol of 200 mg/dl or more, duration of diabetes of 7 years or more, physical activity of less than four days, not self-monitoring blood glucose, types of antidiabetic medication and presence of comorbidity were significantly associated with poorer glycemic control.

The finding from this study suggests that patients with the high WHR had higher odds of having poorly controlled blood glucose. The study conducted in Malaysia also determined that an increasing central obesity was significantly associated with poor HbA1c control [31]. Another study conducted at McGill University-affiliated outpatient clinics were also pointed that for each standard deviation (0.08 unit) increase in WHR there was an approximately 0.35% higher HbA1C [24].

In addition to this, research conducted in Japan, North America and Iran also indicated that abdominal obesity is an important factor in the diabetes control where they have revealed the association between glycemic control and waist circumference [32–34]. Obesity increases the secretion of Non Esterified Fatty Acids (NEFAs) from adipose tissue. NEFAs is associated with insulin resistance [35, 36]. This might be a reason for poor glycemic control in obese patients with diabetes mellitus. Patients with total cholesterol of 200 mg/dl or more were more likely to have poor glycemic control compared to patients with the total cholesterol of less than 200 mg/dl.

A study conducted in San Diego has also demonstrated that patients with total cholesterol level ≥ 200 mg/dl had a higher mean A1C (8.0%) than those with lower total cholesterol level, A1C 7.5% [37]. Similarly, studies conducted in Ethiopia, at Ambo hospital and Tikur Anbessa Specialized Hospital, revealed that patients who had hyperlipidemia were at a more risk of developing poor glycemic control than patients with no comorbidities [19, 21]. This could be because of elevated triglyceride as part of total cholesterol, which results in increasing free fatty acid, which intern related to inflammation and eventually result in insulin resistance or impaired cell function [38].

Duration since diagnoses of diabetes was also another predictor of poor glycemic control.

Patients who had diabetes for the longer duration (greater than 7 years) were more likely to have poor glycemic control. A study conducted in Hawaii (2006) also indicated that patients who had diabetes for more than 10 years were more likely to have poor glycemic control than those who had diabetes for 3 years [39]. In line with these results, a study done by Akour et al. (2011) revealed that patients who were diagnosed with diabetes more than ten years ago were more likely to have poor glycemic control compared to those with duration of less than or equal to 10 years [40]. This is may be due to progressive impairment of insulin secretion with time by ß- cell and increase in insulin resistance and a sudden decrease in insulin secretion [41].

In contradiction to these findings, the study done by Nichols et al. (2000) indicated that the duration of the diagnosis of the disease is not a significant factor for glycaemic control, rather there is a poorer metabolic control among the younger age groups [23].

Physical activity was also another predictor of poor glycemic control. A patient envolved in physical activity only for less than three days were more likely to have poor glycemic control compared to those doing the regular physical activity for more than three days. This is in line with the study done in Jordan and Thailand [30, 42]. This might be because of increasing glucose uptake by the working muscle than a muscle at rest, because physical activity increases the blood flow to the muscle and eventually increases the number of insulin receptors, which finally result in increasing insulin sensitivity [43, 44].

In this study respondents who were not self monitored their blood glucose had higher odds of having poorly controlled blood glucose compared to those who self monitored their blood glucose. A study conducted in Jordan also showed that patients who were less adherent to SMBG had poor glycemic control [30]. Like wise another study conducted in Northern California showed that adherence to SMBG was significantly associated with the lower HbA1c level [45]. Moreover, a study conducted in Mekelle city indicated that patient who adherent to SMBG had good glycemic control [18].

A patient who didn’t self-monitor their blood glucose may not consult health care providers frequently as patients who self-monitor their blood glucose. This could contribute to poor glycemic control in patients who didn’t self-monitor their blood glucose. In addition to this patients who didn’t self-monitor their blood glucose may not adjust their antidiabetic medication and modify their eating plan, though the study done in western Kenya reported that there was no any association between adherence to SMBG and glycemic control. This controversy might be related to small sample size (only 164 participants) were included in the study mentioned above. In addition to this, the patients enrolled were aslo patient with poorly controlled HbA1c [46].

Types of antidiabetic medication were associated with glycemic control. Patients on insulin were more likely to have poorly controlled blood glucose than those on metformin alone. This was in line with the study done in India, which showed that patients on insulin alone had higher odds of poor glycemic control [47].

Similarly, a study conducted by Egede et al. found that the odds of having uncontrolled HbA1c were higher in individuals using insulin only compared to patients taking oral hypoglycemic medication only [48]. This might be due to patients who were prescribed with insulin could have more severe diabetes and diabetes of a longer duration.This was largely contributed by the fact that -cell function worsened as the duration of diabetes increased from the time of diagnosis through follow up [49]. Similarly, patients on metformin plus glibenclamide have a higher probability of poor glycemic control compared to a patient on metformin alone. This could be due to drug therapy problem. For instance the study done in Indonesia showed that the number of medications signifcantly predicted the number of drug related problem [50].This drug related problem in turn affect blood glucose control. But, this study didn’t address factors related to drug therapy problems.

Patients who have one or more comorbidity have higher odds of having poor glycemic control. The study conducted in India revealed that the presence of diseases such as coronary heart disease, neuropathy, retinopathy, renal failure and neurological disorders was associated with poor control of diabetes [51]. Similarly, a study done in Mekelle town northern, found that patient who had hypertension as one part of comorbidity were more likely to be poorly controlled [18]. The reason for comorbidity as a predictor of poor glycemic control might be due to poor adherence of the patients to the medication because of additional medication for the comorbidity might be increasing the pill burden to the patient.

### Strength and limitation of the study

#### The strength of the study

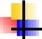
Since this study was case-control its strength of identifying the predictors of poor glycemic is better than cross-sectional study done in Ethiopia previously.

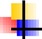
The sample size of this study was larger than many studies done in Ethiopia previously.

#### Limitation of the study

As some parts of the questionnaire depended on the memory of respondents may have resulted in recall bias. Patients with newly diagnosed type 2 diabetes were not included. It was done only among type 2 diabetes patients who were on follow up at outpatient clinic which may not be representative of the overall type 2 diabetes population. HbA1c was not used due to unavailability in the hospital.

## Conclusion

Lack of self-monitoring blood glucose, the presence of comorbidity, longer duration of diabetes mellitus, physical activity of 3 days or less, a total cholesterol level of 200 mg/dl or more, high waist to hip ratio, being on insulin or a combination of metformin with glibenclamide were the independent predictors of poor glycemic control. The health care provider have to encourage the patients during every visit to do 30 min of physical activity.

## Data Availability

The data set is available with authors and can be obtained from the corresponding author up on reasonable request.

## Abbreviations

AOR: Adjusted Odds Ratio
BSc: Bachelor of Science
JUMC: Jimma University Medical Center
